# Electronic Peculiarities of a Self-Assembled M_12_L_24_ Nanoball (M = Pd^+2^, Cr, or Mo)

**DOI:** 10.3390/molecules24040771

**Published:** 2019-02-21

**Authors:** Roxana Mitzayé del Castillo, Roberto Salcedo, Ana Martínez, Estrella Ramos, Luis Enrique Sansores

**Affiliations:** 1 Departamento de Física, Facultad de Ciencias, Universidad Nacional Autónoma de México, Circuito Exterior s/n, Ciudad Universitaria, Coyoacán, CDMX 04510, Mexico; 2 Instituto de Investigaciones en Materiales, Universidad Nacional Autónoma de México, Circuito Exterior s/n, Ciudad Universitaria, Coyoacán, CDMX 04510, Mexico; salcedo@unam.mx (R.S.); martina@unam.mx (A.M.); eramos@iim.unam.mx (E.R.); sansores@unam.mx (L.E.S.)

**Keywords:** metal-organic frameworks, catalysis, hardness, organometallic

## Abstract

We use molecular mechanics and DFT calculations to analyze the particular electronic behavior of a giant nanoball. This nanoball is a self-assembled M_12_L_24_ nanoball; with M equal to Pd^+2^; Cr; and Mo. These systems present an extraordinarily large cavity; similar to biological giant hollow structures. Consequently, it is possible to use these nanoballs to trap smaller species that may also become activated. Molecular orbitals, molecular hardness, and Molecular Electrostatic Potential enable us to define their potential chemical properties. Their hardness conveys that the Mo system is less reactive than the Cr system. Eigenvalues indicate that electron transfer from the system with Cr to other molecules is more favorable than from the system with Mo. Molecular Electrostatic Potential can be either positive or negative. This means that good electron donor molecules have a high possibility of reacting with positive regions of the nanoball. Each of these nanoballs can trap 12 molecules, such as CO. The nanoball that we are studying has large pores and presents electronic properties that make it an apposite target of study.

## 1. Introduction

Nanoballs are giant hollow molecules similar to biological giant hollow structures. The interior of their shell can be highly functionalized and thus interact efficiently with species such as DNA (in a way that is impossible for conventional compounds). Within the shells of these nanoballs, it is possible to store molecules [1,2,3], and they can also be considered to be Metal Organic Frameworks (MOFs). MOFs represent a new class of materials with exciting properties [4,5]. They are highly symmetric [6], have high porosity [7,8], and, in some cases, manifest excellent electrical conductivity [9]. These properties make these systems of interest to different fields of research, for example, molecular recognition, efficient hydrogen production, desulfurization, the capture of CO_2_, and catalysis [10,11,12,13,14,15,16,17,18,19].

**Figure 1:**
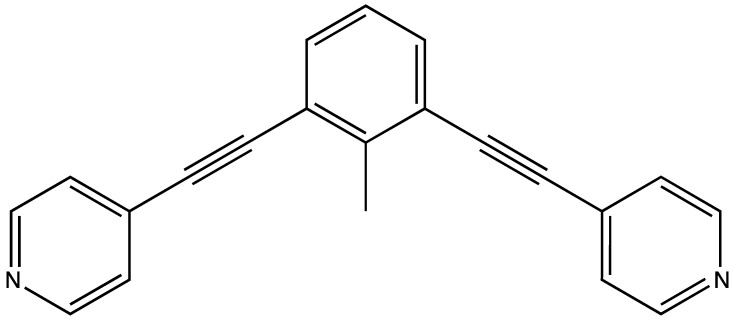
The molecular formula for 4-ethynilpyridine.

Nanoballs have modular chemical arrangements that can be designed in advance in order to obtain systems with particular chemical or physical properties. Their specific application depends on the intrinsic characteristics of each case. Coordination-driven self-assembly is a new synthesis strategy for supramolecular species, which is useful when preparing these frameworks [20,21,22]. This experimental methodology aims to achieve the association of several fragments, which can either be similar or different. These fragments comprise repeated sequences that make up the new complex substance. Several organic molecules exist, which can be used as links in a large chain or as bricks in a polyhedral structure with a planar shape. 4-ethynilpyridine represents an example of this and is presented in Figure 1 [23].

One of the most important characteristics of these materials is their porosity. Several nanoballs have been designed to their potential as a host for other molecules [24]. It would, therefore, be advantageous to design and activate large cages to trap species. Nanoballs can be used to store chemical species or for catalytic purposes [25,26]. It is possible to engineer nanoballs in such a way that certain characteristics (conductivity and reactivity, for example) become paramount. The specific combination of metal ions (or atoms) and struts may form a determined sequence, with a particular geometry and high porosity. In this sense, a precise structure can be designed for a particular purpose. In previous work, Tominaga et al. [22] reported the synthesis of a self-assembled M_12_L_24_ coordination nanoball, with a large hollow coordination cage. The shell of the cage contained 12 metals and 24 ligands and had a spherical shape, with cuboctahedron symmetry. They used Pd^+2^ ions to form the M_12_L_24_ spherical complex and the fragment from Figure 1 in the synthesis of this 24-fold endohedral functionalization that had a sizeable hollow coordination cage [22]. The importance of this nanoball is that it is functionalized, in a way similar to biological large hollow structures (such as spherical viruses) that have a highly functionalized shell interior to ensure efficient interaction with different substances stored within the shells [27,28].

In order to analyze the properties of these types of structures, it is essential to comprehend the electronic structure. In the present work, we theoretically study the nanoball reported by Tominaga et al. (12 ions of Pd^+2^), and we proposed two other complexes based in Pd^+2^. In order to devise similar systems, but neutral ones, we study the structure and electronic properties of similar nanoballs with either Cr or Mo, rather than Pd^+2^. It is well known that these two transition metals can form neutral coordination compounds [29,30,31,32] and planar structures [33,34,35] that allow these nanoballs to exist.

In this paper, we describe these new nanoballs and analyze their electronic properties. This information will be useful for the creation of very tangled molecules [36,37], molecular machines [38,39], etc. The nanoball that we are studying has large pores and presents electronic properties that make it an apposite target of study.

## 2. Computational Details

Starting conformations for geometry optimization were prepared according to experimental information of similar compounds reported before. All the steps that we followed are included as Supplementary Materials. The optimization process for nanoball structures was carried out using the Gaussian 09 computational package (Gaussian, Inc, Carnegie Mellon University, Pittsburgh-PA, USA) [40]. We used a Universal Force Field (UFF) to optimize geometries at a Molecular Mechanics level [41,42]. UFF is a handy tool, and reports have presented good results for organometallic frameworks, such as C_60_ fullerene derivatives, and other intertwined coordination cages [43,44]. Following the optimization process, one single point was calculated using the Turbomole Computational Package (TURBOMOLE GmbH, University of Karlsruhe, Karlsruhe, BW, Germany) [45]. This calculation was performed by applying the Density Functional Theory with a dispersion correction BJ [46] (DFT-D3) with the functional PW6B95 [47] and the def-SVP basis set [48]. This method has been successfully used in the past to describe metal-organic frameworks [49]. For metal atoms, Effective Core Potentials (ECPs) were used (Cr/ecp-10-mdf, Mo/def2-ecp, and Pd/def2-ecp). These ECPs include scalar relativistic approximations [50,51].

## 3. Results and Discussion

The primary purpose of this investigation is to study M_12_L_24_ spherical complexes. The structures used in this study present an endohedral functionalization of a large spherical hollow coordinate cage. The shell of the nanoball structure contains 12 metal atoms (Cr and Mo) and 24 ligands (L). Each ligand has a bis (4-pyridyl)-CH_3_ bent framework with two acetylene spacers. The 24 ligands assemble to form a spheroidal shape that connects to the metal atom.

**Figure 2:**
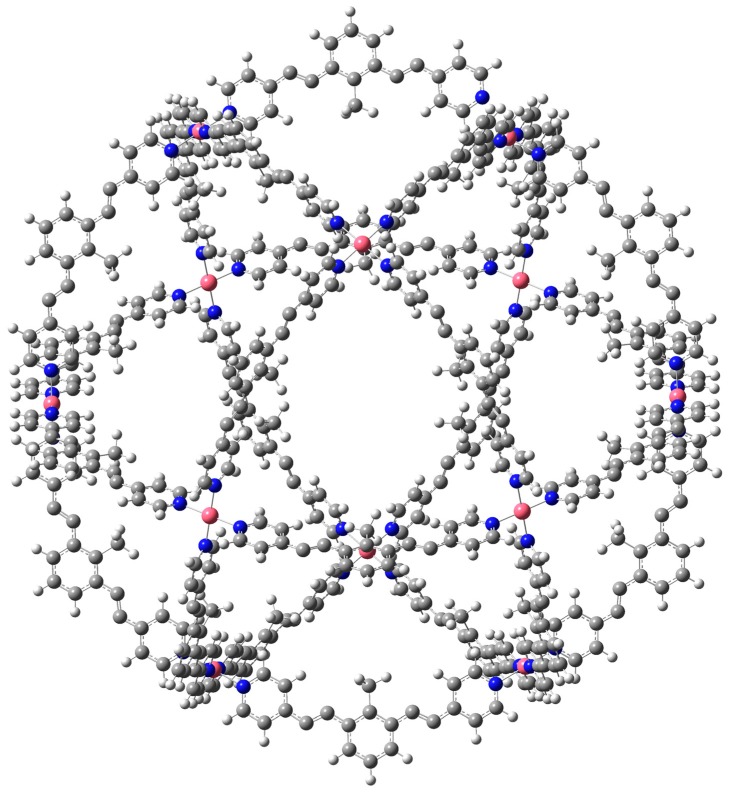
Optimized structure of (Pd^+2^)_12_L_24_. L is bis (4-pyridyl)-CH_3_ bent framework with two acetylene spacers.

**Figure 3:**
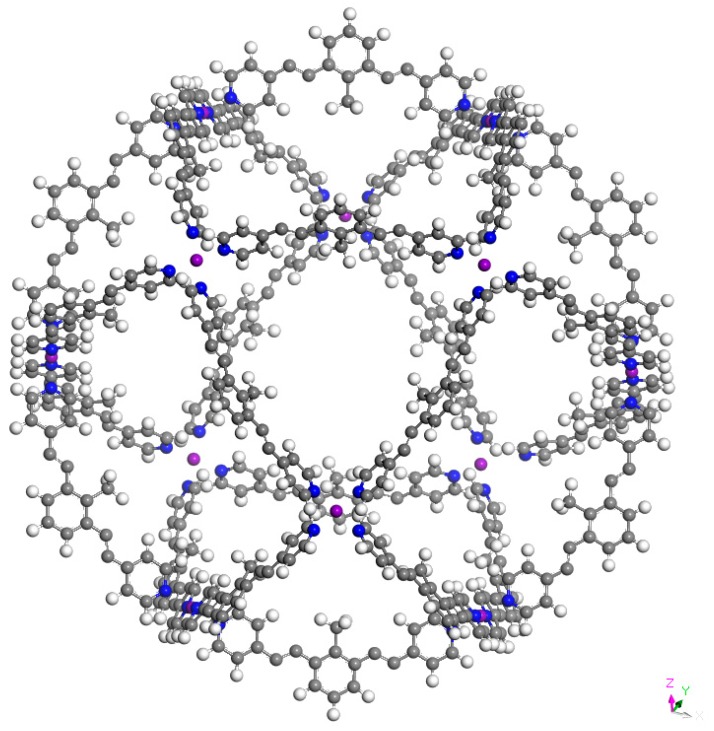
Optimized structure of Cr_12_L_24_. L is bis (4-pyridyl)-CH_3_ bent framework with two acetylene spacers.

**Figure 4:**
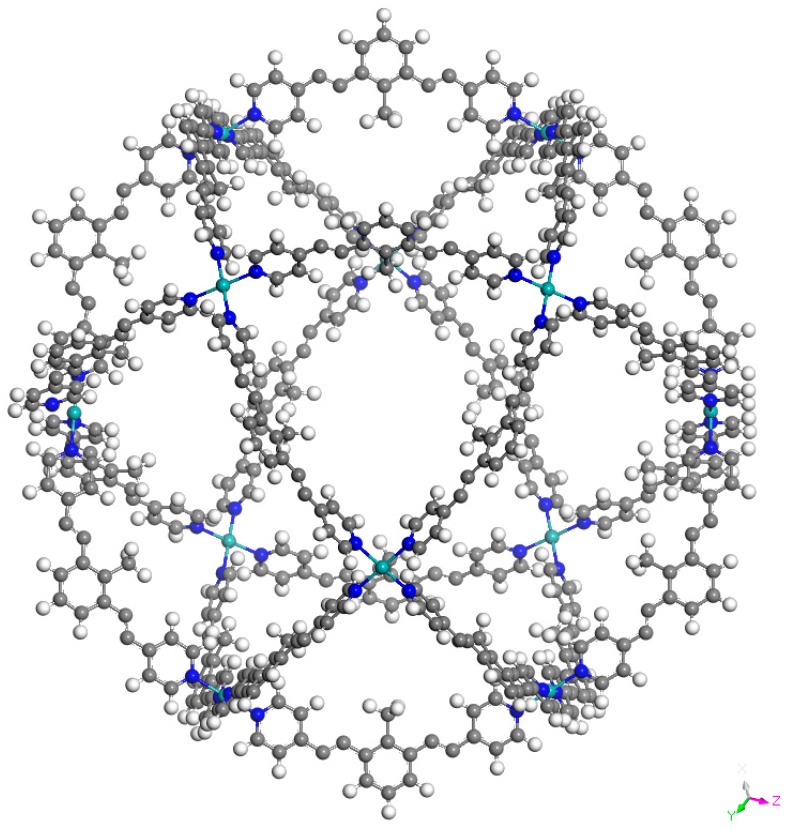
Optimized structure of Mo_12_L_24_. L is bis (4-pyridyl)-CH_3_ bent framework with two acetylene spacers.

Figure 2, Figure 3 and Figure 4 present the optimized structures of the nanoballs used in this work, with Pd^+2^, Cr, and Mo respectively. A nanoball previously proposed by Tominaga et al. [22] inspired our design. There is a similarity between the linking molecules and symmetry. 12 metal atoms are linking the 24 ligands (reaching a total of 1000 atoms). As explained previously, in this molecule the metal atoms are Pd^+2^, Cr, or Mo. These metals were selected as they form strong coordination covalent bonds with the terminal nitrogen atoms from the pyridine ring, and they have a zero oxidation state. These are thus neutral molecules. Consequently, all possible interactions with these species are expected to manifest weak dispersion and no full atomic charge attractions.

It is important to note that optimized structures present sp carbons of the acetylene fragment of the ligand that has a bent conformation. We performed single point calculations of the nanoballs with a linear conformation of the acetylene fragment, and the energy indicates that this is less stable. The characteristics of the nanoball request the bent conformation due to the molecular stress. In this case, the presence of linear sp carbons is less important, since the reduction of the molecular stress when the acetylene is bent contributes more to the stability.

M_12_L_24_ supramolecular cages have a diameter of 35.9, 36.0, and 36.1 Å for Pd^+2^, Cr, and Mo, respectively. This is the distance between two metal atoms over one diameter of the sphere. The acetylene spacer enlarges the diameter of the cage independently of the metal atom, concurring with previous experimental results [22]. It is important to conserve the acetylene spacer because it prevents the nonplanar configuration of the ligand and also contributes to preserving spherical symmetry. The diameter of C_60_ fullerene (6.8 Å approximately) is almost five times smaller than the diameter of this nanoball. This means that it is possible for a fullerene to reside within the nanoball, increasing the solubility of fullerene in water. We assume that the nanoball is soluble in water, as the experimental results for the nanoball with Pd^+2^ have been reported for this solvent.

**Figure 5:**
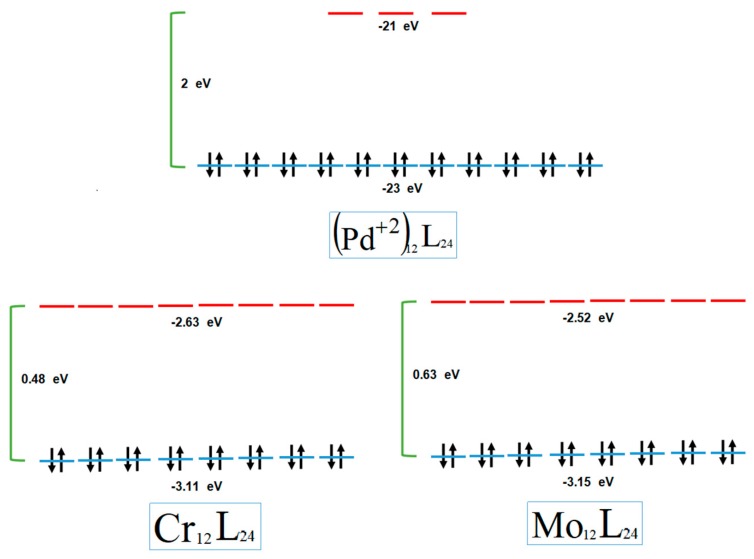
Eigenvalues of the nanoball with Pd^+2^, Cr, and Mo. The HOMO–LUMO (highest occupied molecular orbitals, lowest occupied molecular orbitals) gap is also included.

In the three systems, frontier orbitals are highly degenerate (see Figure 5). For the system with Cr and Mo, there are 12 quasi-degenerated orbitals that correspond to the highest occupied molecular orbitals (HOMOs), and the lowest unoccupied molecular orbitals (LUMOs) are also 12 quasi-degenerated orbitals. Meanwhile, the Pd^+2^ has 11 HOMOs quasi-degenerated orbitals and 3 LUMOs quasi-degenerated orbitals. 

Conceptual Density Functional Theory, based on theoretical reactivity indexes, is a powerful tool to study organic reactivity [52,53]. In particular, chemical hardness (η) and Mulliken electronegativity (χ) are very useful, since hardness is related to the stability and Mulliken electronegativity is a measure of the resistance to electron density loss. These two quantities are defined as Equations (1) and (2):

η = I − A(1)

χ = (I + A)/2(2)

Using Kohn–Sham formalism and Koopmans’ theorem, ionization energy and electron affinity can be approached by the absolute values of the frontier HOMO and LUMO energies, respectively. Therefore, the HOMO–LUMO gap is associated with a chemical. Concurring with ideas from Parr et al., it appears that systems are more reactive when hardness is diminished. Considering our systems, the HOMO–LUMO gap is 2 eV for Pd^+2^, 0.48 eV for the Cr nanoball, and 0.63 eV for the system with Mo. The Pd^+2^ and Mo systems are less reactive than the Cr system. The eigenvalues in each case are similar, but HOMOs from the Mo nanoball manifest less energy than corresponding orbitals from the Cr system. This means that the electron transfer from the system with Cr to other molecules will be more favorable than for the system with Pd^+2^ or Mo. For the nanoballs studied here, the values of χ are 22, 2.87, and 2.83 eV (for a system with Pd^+2^, Cr, and Mo, respectively). As expected, the system with Pd^+2^ is very resistant to electron density loss, since it is positively charged. With Cr and Mo, both systems present similar value for the electronegativity.

Table 1 shows the total energies (*E_total_*) and the Binding Energies (*E_binding_*) calculated with the following Equation (3):

*E_binding_ = E_total_ − [(24E_ligand_) + (12E_metal_)]* (3)

The E_ligand_ = −3425.461 eV, E_Pd_^+2^ = −2315.861 eV, E_Cr_ = −1838.547 eV, and E_Mo_ = −24,798.535 eV calculated with the same theory level.

**Figure 6:**
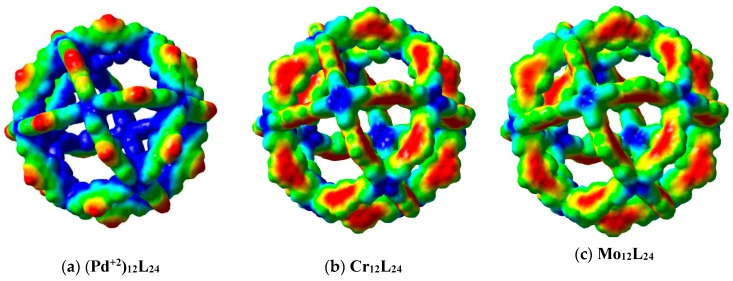
(**a**–**c**) Molecular electrostatic potential mapped onto electron density for nanoballs. Red/Blue colors represent negative/positive values.

The Molecular Electrostatic Potential is a useful tool for analyzing the electronic properties of these large pore nanoballs. Results are presented in Figure 6. The negative electrostatic potential is in red, whereas positive is reported in blue.

From the Electrostatic Potential of the Pd^+2^ nanoball, it is observed that there is a strong electronic polarization, indicating that the positive part of the electrostatic potential is inside the nanoball and the metal atoms. So, negatively charged compounds can be attracted to the interior of the nanoball, due to the polarization.

Of the other systems, with neutral metals (Cr and Mo), it is observed that the electrostatic potential is quite similar. Metal atoms have a positive contribution to the electrostatic potential surface and could attract negative compounds. Meanwhile, the ligands present negative electrostatic potential and are more related to catching positive compounds. It is apparent that some pores have positive walls, whereas others have negative frontiers.

The electrostatic potential allows us to see where the sites with the greatest possibility of reactivity are located. The interactions will be defined by the molecules that are approaching; if the molecules are positive, they will feel a force of attraction towards the ligands, and if the molecules are negative, they will feel a force of attraction to the metals.

Moreover, Molecular Electrostatic Potential means that good electron donor molecules have a high possibility of reacting with a positive region of the nanoball. For example, CO or NO will interact with Cr or Mo. If this were the case, each of these nanoballs would be able to react with 12 of these molecules, and therefore each nanoball would be able to trap 12 molecules. These electronic properties make these important nanoballs systems with many possible applications. Molecules that approximate to these nanoballs will be trapped.

## 4. Conclusions

The extraordinarily large cavity within these nanoballs may be highly functionalized, in a way that is similar to giant hollow biological structures. Interaction with different molecules may be possible, depending on the molecule that is approximated. It is apparent that they would be able to trap smaller species that could then also be activated.

Hardness values affirm that the Pd^+2^ and Mo systems are less reactive than the Cr system. Eigenvalues indicate that electron transfer from the system with Cr to other molecules is more favorable than for the system with Mo.

As the electrostatic potential is both positive and negative, it follows that molecules which interact with these nanoballs may also become functionalized by either an electron transfer from the nanoball to the molecule or from the molecule to the nanoball.

Molecules that are good electron donors will react with metal atoms from nanoballs that are positive. Consequently, each of these nanoballs would be able to react with 12 of these molecules. These electronic properties make these nanoballs an important target of study.

Each of these nanoballs can react with 12 molecules, such as CO. Thus each nanoball is able to trap 12 molecules. Molecules that approximate to these nanoballs will thus be trapped. The nanoballs presented here have large pores and present electronic properties that make it an apposite target of study.

## Figures and Tables

**Table 1:** Binding energies and total energies of the nanoballs.

System	Binding Energy (eV)	Total Energy (eV)
Pd^+2^	−3.06	−636,353.1585
Cr	−2.05	−623,010.6125
Mo	−1.39	−617,264.9439

## Supplementary Materials

The construction of the nanoballs are available online. Also, it is available the structure of the nanoball, in format xyz, and the ligand structure.
