# Erratum: Del Castillo, R.M., et al. Electronic Peculiarities of a Self-Assembled M_12_L_24_ Nanoball (M = Pd^+2^, Cr, or Mo). *Molecules* 2019, *24*, 771

**DOI:** 10.3390/molecules25020267

**Published:** 2020-01-09

**Authors:** Roxana Mitzayé del Castillo, Roberto Salcedo, Ana Martínez, Estrella Ramos, Luis E. Sansores

**Affiliations:** 1 Departamento de Física, Facultad de Ciencias, Universidad Nacional Autónoma de México, Circuito Exterior s/n, Ciudad Universitaria, Coyoacán, CDMX 04510, Mexico; 2 Instituto de Investigaciones en Materiales, Universidad Nacional Autónoma de México, Circuito Exterior s/n, Ciudad Universitaria, Coyoacán, CDMX 04510, Mexicomartina@unam.mx (A.M.); eramos@iim.unam.mx (E.R.); sansores@unam.mx (L.E.S.)

The author wishes to make the following correction to this paper [1]. In Table 1, the units of the second column are Hartrees, replace the following:

**Table 1:** Binding energies and total energies of the nanoballs.

System	Binding Energy (eV)	Total Energy (eV)
Pd^+2^	−3.06	−636353.1585
Cr	−2.05	−623010.6125
Mo	−1.39	−617264.9439

with

**Table 1:** Binding energies and total energies of the nanoballs using E_Pd_^+2^ = −3425.461 eV, E_Cr_ = −2315.861 eV, E_Mo_ = −1838.547 eV, and E_ligand_ = −24,798.535 eV.

System	Binding Energy (Hartrees)	Total Energy (eV)
Pd^+2^	−3.06	−636353.1585
Cr	−2.05	−623010.6125
Mo	−1.39	−617264.9439

The conclusions continue to remain the same. The authors would like to apologize for any inconvenience caused to the readers by these changes.
