# Supplementary material for: Electronic Peculiarities of a Self-Assembled M12L24 Nanoball (M = Pd+2, Cr, or Mo)
Source: Molecules. 2019 Feb 21;24(4):771. doi: 10.3390/molecules24040771 (PMC6412375; doi:10.3390/molecules24040771)
Supplement: Supplementary file 1 [file molecules-24-00771-s001.zip › SupplemetaryInformation/Supporting_Information.docx]

Article

Electronic peculiarities of a Self-Assembled M_12_L_24_ nanoball (M=Pd^+2^, Cr, or Mo)

Supporting Information

Roxana M. del Castillo^1^, Roberto Salcedo^2^, Ana Martínez^2^, Estrella Ramos^2^, Luis E. Sansores^2^

^1^ Departamento de Física, Facultad de Ciencias, Universidad Nacional Autónoma de México, Circuito Exterior s/n, Ciudad Universitaria, Coyoacán, 04510, CDMX, México

^2^ Instituto de Investigaciones en Materiales, Universidad Nacional Autónoma de México, Circuito Exterior s/n, Ciudad Universitaria, Coyoacán, 04510, CDMX, México

***** Correspondence: [roxanadelcastillo@ciencias.unam.mx](mailto:roxanadelcastillo@ciencias.unam.mx)

Received: date; Accepted: date; Published: date

S1. Additional Information about the construction of the Nanoball.

| 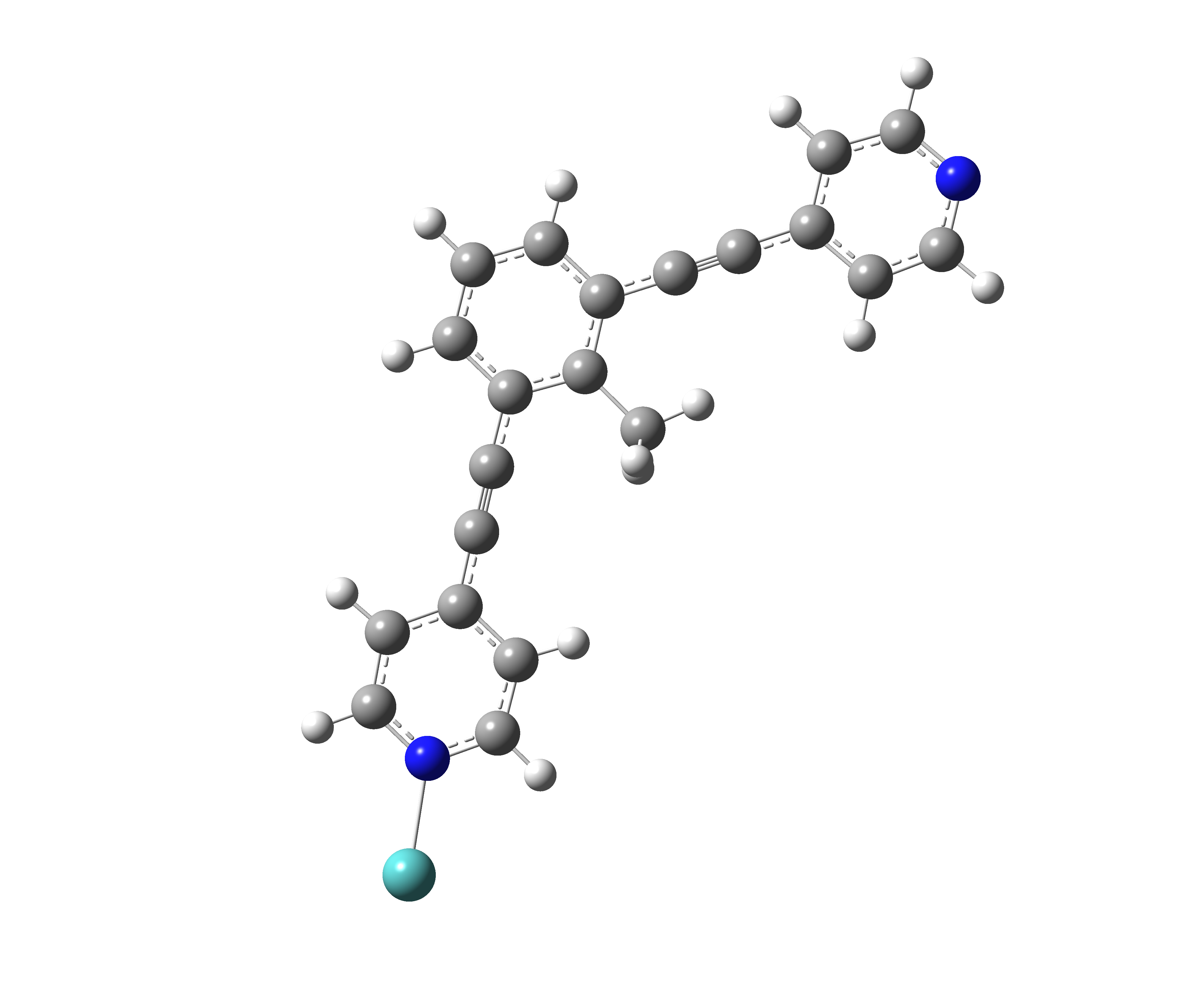  Step 1. Optimize of the Ligand (bis(4-pyridyl)-CH_3_) with the sp carbons on the acetylene fragments and a metal atom. | 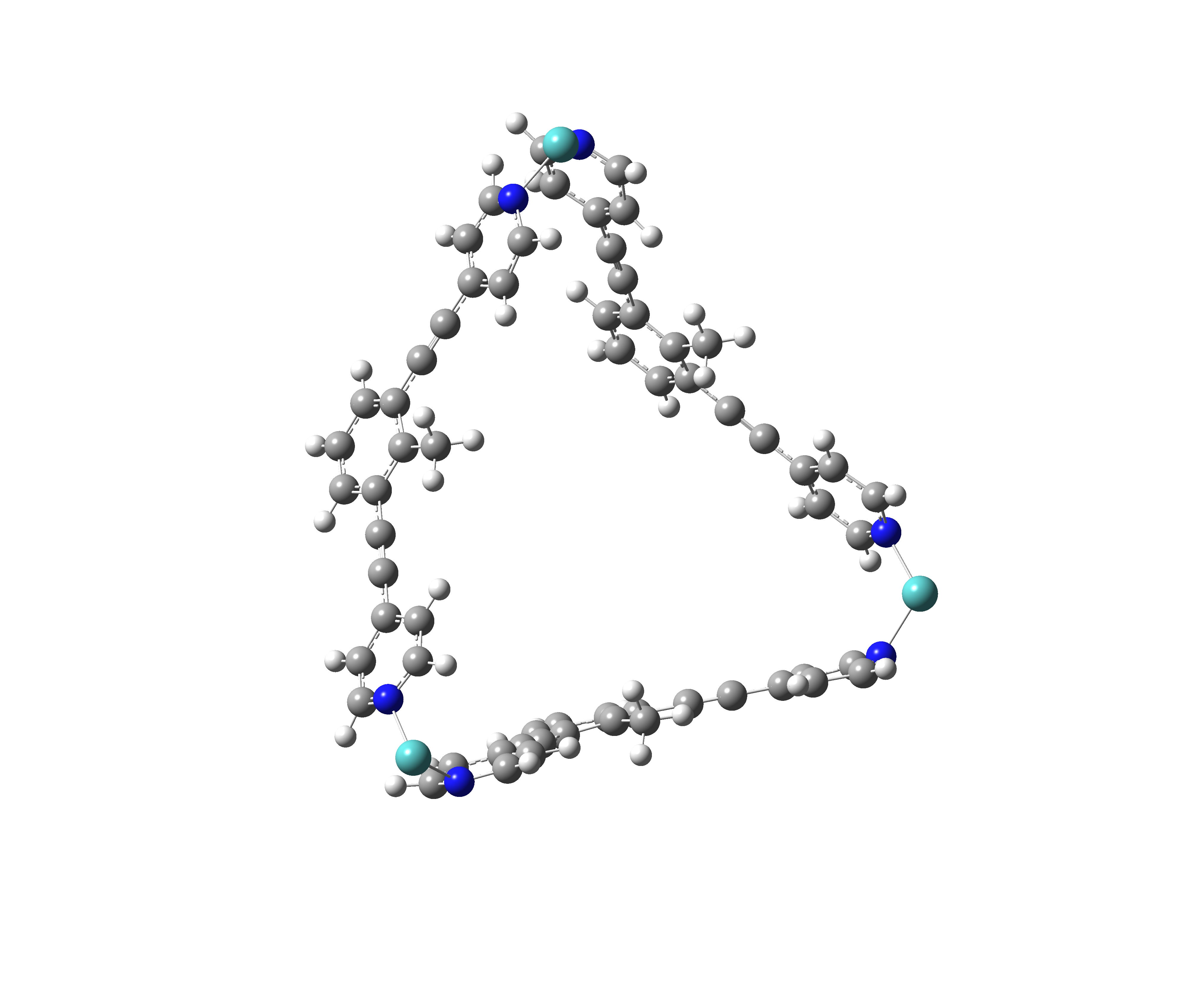  Step 2. Optimize of 3 Ligands (bis(4-pyridyl)-CH_3_) bonding with 3 metal atoms. |
| --- | --- |
| 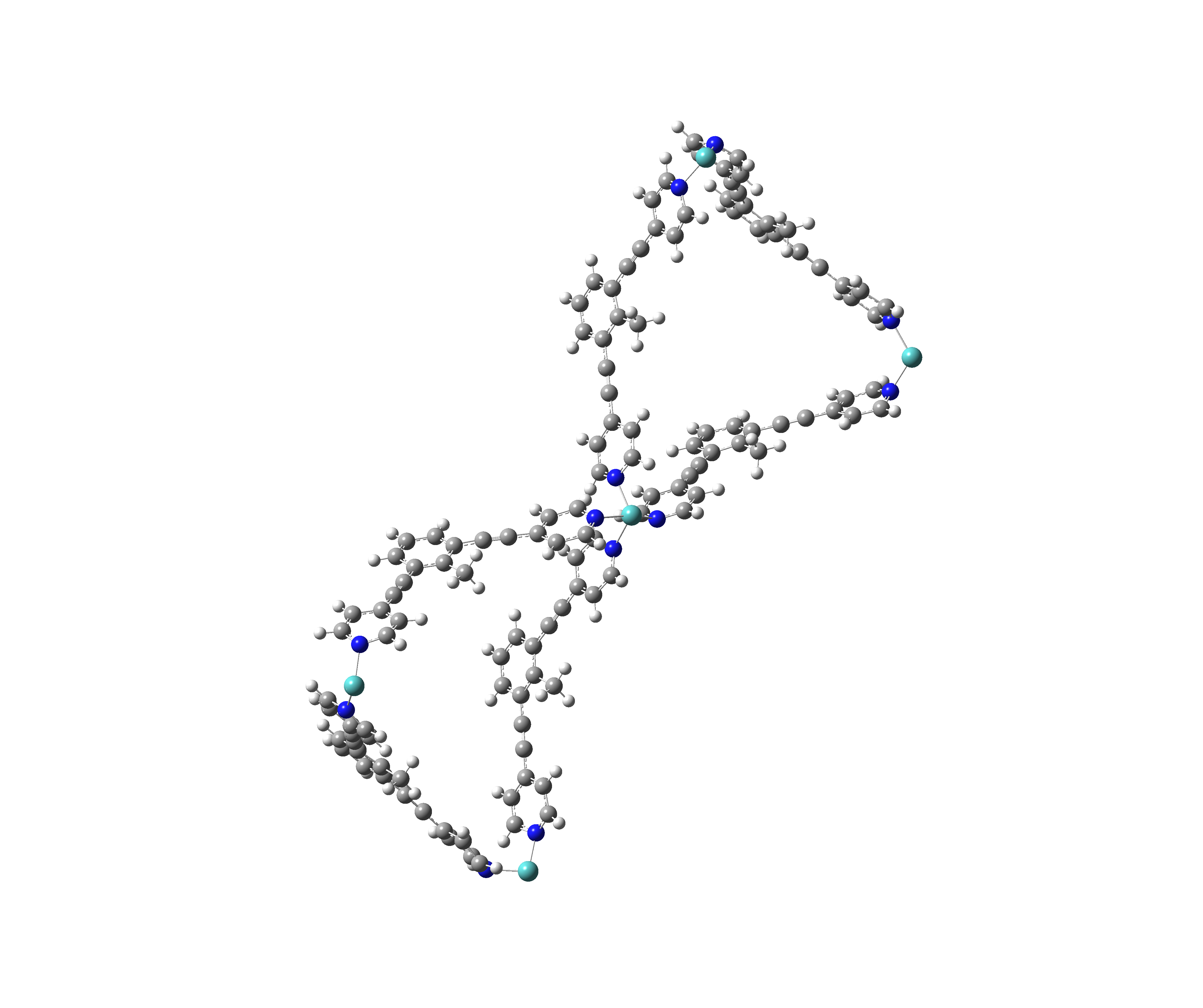  Step 3. Optimize of 6 Ligands (bis(4-pyridyl)-CH_3_) bonding with 5 metal atoms. | 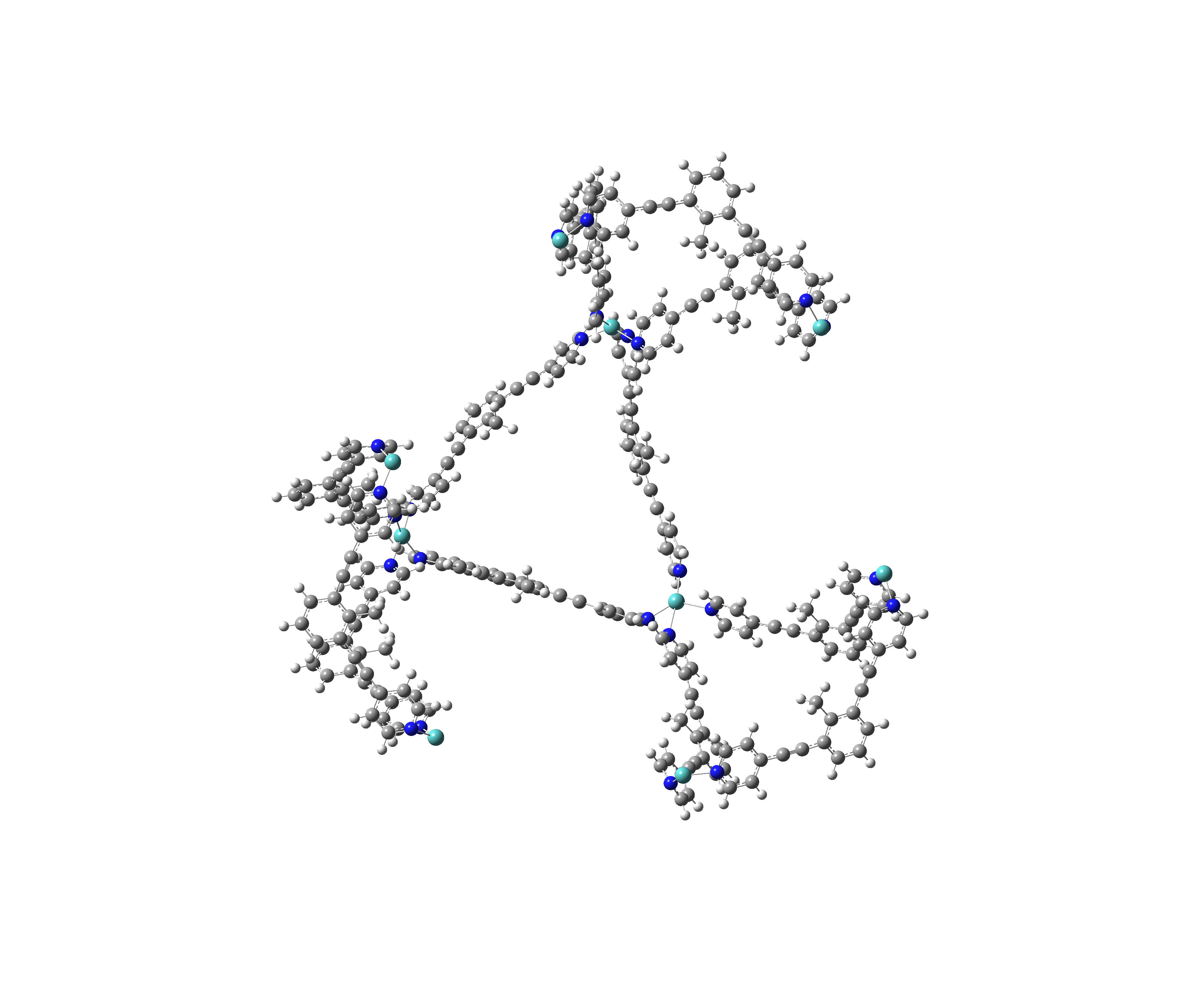  Step 4. Optimize of 12 Ligands (bis(4-pyridyl)-CH_3_) bonding with 9 metal atoms. |
| 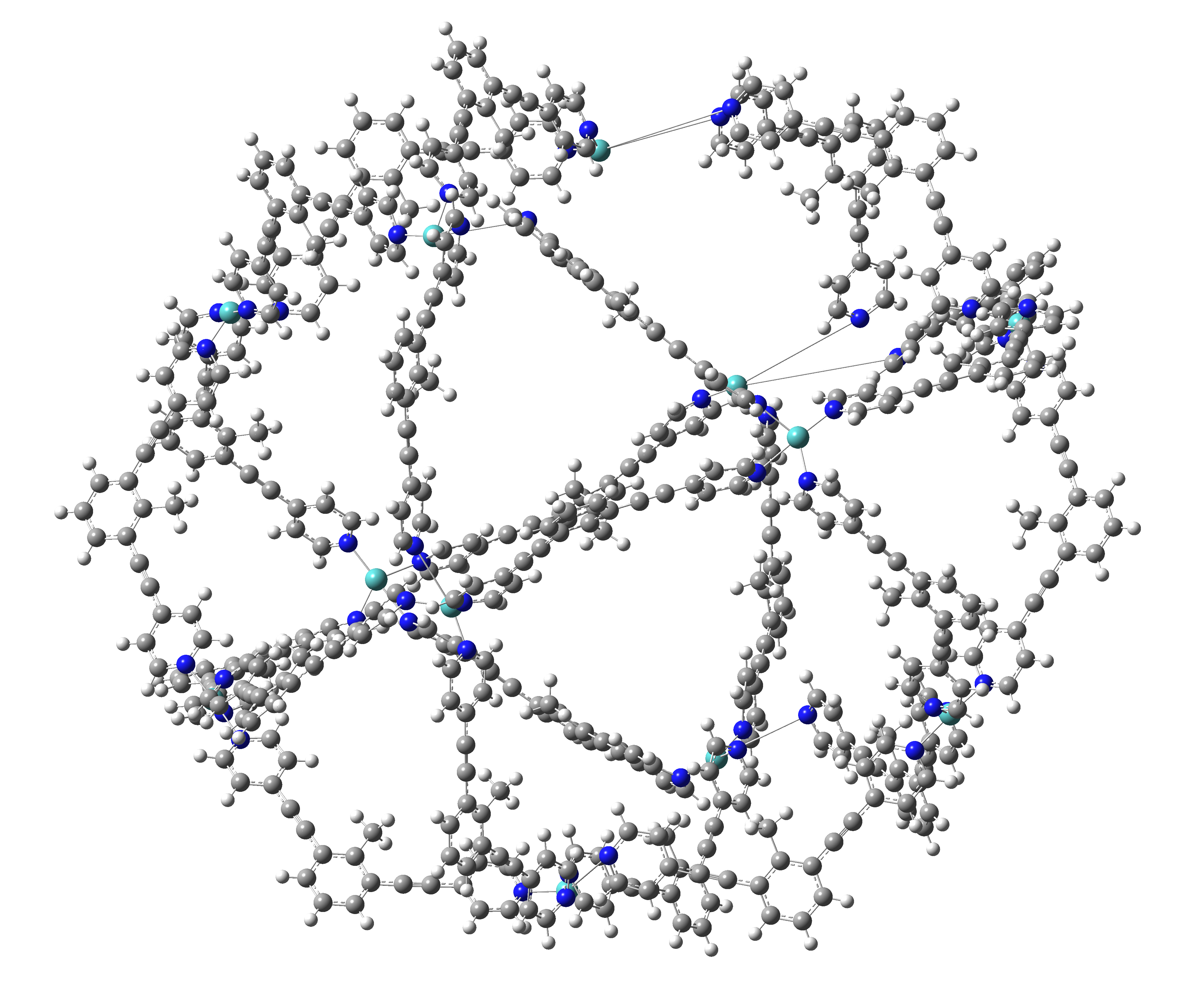  Step 5. Join two-part obtained in step 4, 24 Ligands (bis(4-pyridyl)-CH_3_) bonding with 12 metal atoms. | 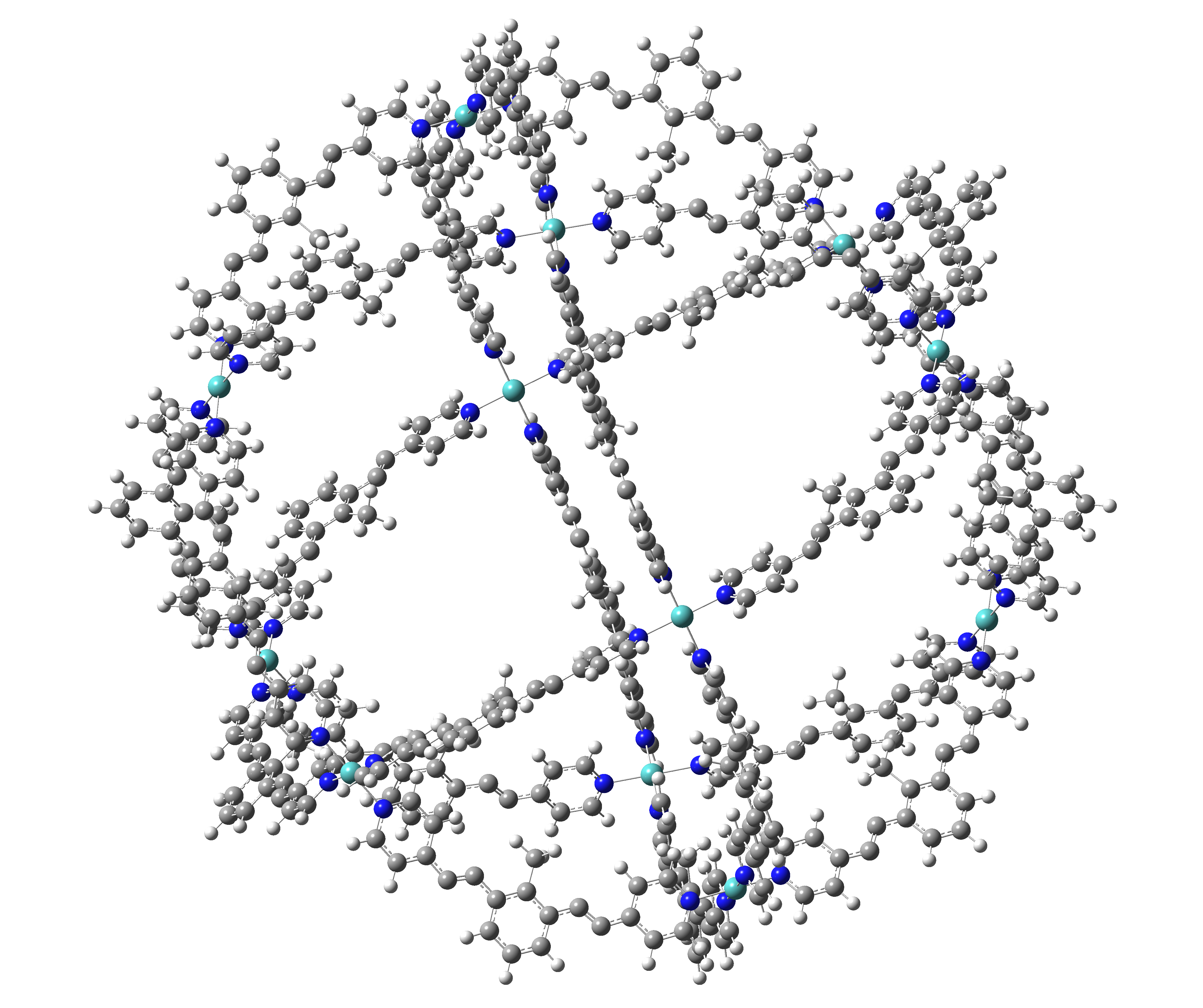  Step 6. Optimize the Nanoball, 24 Ligands (bis(4-pyridyl)-CH_3_) bonding with 12 metal atoms. The acetylene fragment of the ligand that has a bent conformation |

The file **Ligand.xyz** has the optimized structure of the Ligand (bis(4-pyridyl)-CH_3_), which is the step on and

the file **Nanoball_Mo.xyz** has the final structure of the Mo_12_L_24_.
